# Protocol for a systematic review on the extent of non-publication of research studies and associated study characteristics

**DOI:** 10.1186/2046-4053-2-2

**Published:** 2013-01-09

**Authors:** Susan Portalupi, Erik von Elm, Christine Schmucker, Britta Lang, Edith Motschall, Guido Schwarzer, Isabel T Gross, Roberta W Scherer, Dirk Bassler, Joerg J Meerpohl

**Affiliations:** 1 German Cochrane Centre, Institute of Medical Biometry and Medical Informatics, University Medical Center Freiburg, Freiburg, Germany; 2 Cochrane Switzerland, IUMSP, University Hospital Lausanne, Lausanne, Switzerland; 3 Institute of Medical Biometry and Medical Informatics, University Medical Center Freiburg, Freiburg, Germany; 4 Center for Pediatric Clinical Studies, University Medical Center Tuebingen, Tuebingen, Germany; 5 US Cochrane Center, John Hopkins Bloomberg School of Public Health, Baltimore, MD, USA

**Keywords:** Publication bias, Selective reporting, Abstract(s), Research ethics committees, Trial registration, The OPEN Project

## Abstract

**Background:**

Methodological research has found that non-published studies often have different results than those that are published, a phenomenon known as publication bias. When results are not published, or are published selectively based on the direction or the strength of the findings, healthcare professionals and consumers of healthcare cannot base their decision-making on the full body of current evidence.

**Methods:**

As part of the OPEN project (http://www.open-project.eu) we will conduct a systematic review with the following objectives:

1. To determine the proportion and/or rate of non-publication of studies by systematically reviewing methodological research projects that followed up a cohort of studies that

a. received research ethics committee (REC) approval,

b. were registered in trial registries, or

c. were presented as abstracts at conferences.

2. To assess the association of study characteristics (for example, direction and/or strength of findings) with likelihood of full publication.

To identify reports of relevant methodological research projects we will conduct electronic database searches, check reference lists, and contact experts. Published and unpublished projects will be included. The inclusion criteria are as follows:

a. RECs: methodological research projects that examined the subsequent proportion and/or rate of publication of studies that received approval from RECs;

b. Trial registries: methodological research projects that examine the subsequent proportion and/or rate of publication of studies registered in trial registries;

c. Conference abstracts: methodological research projects that examine the subsequent proportion and/or rate of full publication of studies which were initially presented at conferences as abstracts.

Primary outcomes: Proportion/rate of published studies; time to full publication (mean/median; cumulative publication rate by time).

Secondary outcomes: Association of study characteristics with full publication.

The different questions (a, b, and c) will be investigated separately. Data synthesis will involve a combination of descriptive and statistical summaries of the included methodological research projects.

**Discussion:**

Results are expected to be publicly available in mid 2013.

## Background

Full information about completed and ongoing clinical studies is the indispensable base for decision-making about medical therapies, treatments, and diagnostic procedures by patients, doctors, and policy-makers. Equally, researchers and research organizations, research ethics committees, governments and health system agencies, courts for social justice, pharmaceutical companies, and all professional groups of the healthcare system are dependent on unbiased information. Methodological research projects have shown that for approximately 40% to 50% of all launched studies, results or reasons for their failure are never published [1-4]. Evidence shows that these unpublished studies do not significantly vary in methodological quality from their published counterparts [5,6]. The lack of publication of completed studies was termed ‘the file drawer problem’ by Rosenthal in 1979 [7].

The frequency of unpublished studies is particularly problematic because several methodological research projects have found that non-published studies often have different results than those that are published, a phenomenon known as publication bias [8]. It has also been found that studies with non-novel or negative findings take longer to reach publication than studies with novel findings or findings in favor of the study arm [9,10]. When results are not published, or are published selectively based on the direction or the strength of the findings, healthcare professionals and consumers of healthcare cannot base their decision-making on the full body of current evidence.

The inability to make evidence-informed decisions impacts the healthcare system at various practical and financial levels. At the individual level, the overestimation of treatment effects caused by publication bias may result in patients receiving treatments that may be more harmful or less efficacious than previously believed. Furthermore, non-publication of studies results in considerable financial investment by funders without any return. Further costs include those incurred by hospitals and medical centers, insurance companies, and individual patients whom all continue to pay for treatments that may not be the most effective or efficient. Although the full extent of financial impact of non-publication of studies is currently unknown, the waste of funds is likely to be high.

In response to these concerns the OPEN Project (To Overcome failure to Publish nEgative fiNdings) was developed with the goal of elucidating the scope of non-publication of studies. The OPEN Project is a 24-month project co-funded by the European Commission under the Seventh Framework Programme. With an international work group composed of key opinion leaders (for detailed information see Appendix), the project will first examine the current evidence on publication bias through a series of systematic reviews such as the one described in this protocol. Second, the OPEN Project will examine current practices by key groups in the field of biomedical research (namely funding agencies, the (pharmaceutical) industry, research ethics committees, research institutions, researchers, trial registries, biomedical journals, regulatory agencies, and benefit assessment agencies), through surveys, interviews, and analysis of current policies and guidelines. These findings will be presented and discussed during an international workshop, which aims to develop recommendations for implementing effective measures to avoid non-publication of studies and related publication bias at all levels.

Non-publication impacts the influence of the results of specific studies as well as of any systematic review in which the individual study could be included. The inability of systematic reviews to include all current evidence may result in misleading conclusions [11]. Previous reviews have been conducted on different aspects of non-publication of studies and publication bias [12-14]. However, some of these reviews were done several years ago, while others are quite specific in scope, focusing only on specific aspects of non-publication of studies and/or publication bias.

Non-publication of studies and publication bias can be revealed through a variety of sources. Accordingly, this review seeks to summarize the current knowledge on non-publication of studies and publication bias by examining cohorts of studies approved by research ethics committees, by examining cohorts of studies registered with trial registries, and finally by examining cohorts of studies that have been initially presented in abstract form at conferences.

### Objectives

This review has two objectives:

1) To determine the proportion and/or rate of non-publication of studies by systematically reviewing methodological research projects that followed-up a cohort of studies that were:

a. approved by research ethics committees, or

b. registered in trial registries, or

c. presented in abstract form at conferences.

2) To assess the association of study characteristics (for example, direction and/or strength of findings) with likelihood of full publication.

## Methods

### Search methods for identification of methodological research projects

To identify the relevant research evidence we will conduct electronic literature searches in the following databases: Ovid Medline (1946 to present), Embase (1980 to present), The Cochrane Library (most current issue), ISI Web of Science (Science Citation Index Expanded [SCI-EXPANDED] 1945-present; Social Sciences Citation Index [SSCI] 1975-present; Arts & Humanities Citation Index [A&HCI] 1975-present; Conference Proceedings Citation Index- Science [CPCI-S] 1990-present; and Conference Proceedings Citation Index-Social Science & Humanities [CPCI-SSH] 1990-present). No language restrictions will be applied. The search strategy was designed with consideration of similar reviews’ strategies and with the support of a librarian/information specialist. The strategy was adapted according to the requirements of each database (for the full search strategy, please see Appendix).

We will also search reference lists of articles retrieved from our database searches to further help us to identify relevant articles, authors, and opinion leaders who might not have been captured in our original search or known to us previously. These individuals will be contacted and asked for further relevant articles.

### Data collection and analysis

#### Selection of methodological research projects

Reports of methodological research projects including a cohort of studies will be selected using a specifically designed full text screening form with predefined inclusion and exclusion criteria (see below). The criteria will be deliberately broad in order to capture as large a scope of biases related to non-publication of studies as possible. Published and unpublished methodological research projects on the fate of a cohort of studies will be included. The inclusion criteria for the individual aspects are as follows:

(1) Methodological research projects of studies approved by research ethics committees (RECs): methodological research projects that examine the subsequent proportion and/or rate of publication of studies that received approval from ethics committees; reports of methodological research projects must provide the number of studies approved and the proportion or rate of approved studies which have been published.

(2) Methodological research projects of studies registered in trial registries: methodological research projects that examine the subsequent proportion and/or rate of publication of studies registered in trial registries; reports of methodological research projects must provide the number of studies registered and the proportion or rate of registered studies which have been published.

(3) Methodological research projects of studies presented as abstracts at a conference or as summary reports: methodological research projects that examine the subsequent proportion and/or rate of full publication of study results which were presented at conferences as abstract or as summary report; reports of methodological research projects must provide the number of abstracts (summary reports) identified and the proportion or rate of abstracts (summary reports) published as full articles.

The included methodological research projects do not need to apply a minimum follow-up to assess time to full publication; however, to calculate the proportion of published studies and/or publication rate, a minimum follow-up of 24 months will be required. Two reviewers will screen the titles and abstracts of all references identified by the literature search. Full text articles will be evaluated for all papers identified as potentially relevant.

#### Data extraction and management

A specifically designed data extraction form is being developed and pilot-tested for each aspect (RECs, trial registries, abstracts). Two reviewers will independently extract all relevant data from eligible studies. Data will be recorded in an Excel spread sheet.

#### Assessment of study quality in included methodological research projects

We will systematically consider the validity and generalizability of the identified evidence provided by each of the methodological research projects by evaluating the following domains:

Sampling: Choice of sampling frame (for example, all abstracts submitted, all accepted as oral presentations, and so on) and sampling method (for example, all abstracts, random sample of abstracts, and so on).

Literature search: Timing of search (for example, how many months after abstracts were presented at a conference was the literature search conducted) and methodology used for identifying full publications (for example, two independent researchers, direct contact with investigators, number and choice of databases, database search for first and/or last author, and so on).

Matching of publications: Methodology used to match retrieved references with studies included in cohort of studies (for example, matching by keywords, title, author names and so on, matching completed by one or more researchers, adjudication if differences, and so on).

Analysis: Adjustment for confounding factors if analysis for factors associated with full publication was done.

Methodological research projects will be categorized as low study quality if the search for publications was likely to miss a substantial number of full publications (for example, if it were only conducted in a single database or if choice of databases is judged to be not appropriate by two reviewers independently). A similar approach will be taken for the other three domains.

### Outcome measures

Our primary outcomes will be the proportion and/or rate of studies published and the time to publication. When possible, pooled proportions of unpublished studies will be calculated. For outcomes pooled, we will explore heterogeneity by standard measures.

The following definitions will be used: Study completion is defined as the last day of follow-up of participants; Time to publication is defined as the interval between study completion and publication. If the end of follow-up is not known (for example for studies presented as abstracts) time to publication will be calculated based on the interval between abstract presentation and subsequent full.

Secondary outcomes include association of study characteristics with full publication. We will evaluate factors that could be associated with the publication of studies, such as direction and/or strength of findings. We plan - if reported or estimable - to collate information on costs/resource use incurred by studies that were not published.

### Unit of analysis issues

The unit of analysis is the methodological research project. At the time of writing this protocol, we do not anticipate any unit of analysis issues.

#### Dealing with missing data

In the instance of missing data, lead researchers of the methodological research projects will be contacted for further information.

#### Assessment of heterogeneity

Heterogeneity for pooled outcome measures will be assessed by standard methods including Chi^2^-test and calculation of the I^2^ value.

#### Assessment of reporting biases

Funnel plots will be used to assess the association between point estimates of log odds ratio (a measure of extent of association between study characteristic and likelihood of publication) and a measure of precision if more than 10 methodological research projects provide necessary information. Funnel plots will be visually assessed and appropriate formal statistical tests following recommendations formulated by Sterne *et al.* will be used to test for asymmetry [15].

#### Data synthesis

The three aspects (research ethics committees, trial registries, and conference abstracts) will be considered separately. Data synthesis will involve a combination of descriptive and statistical summaries of the findings of the included methodological research projects. We will calculate the pooled proportion and/or rate of publication with 95% CI using a random effects model for all included methodological studies by averaging the individually reported publication rates after weighting by the square root of the total number of studies included in each methodological research project. If possible, time to publication will be estimated by survival analyses and depicted in Kaplan Meier curves. If required data are not available, we will simply present proportions at the following time points: 12, 24, 36, 48, 60 months, and so on.

Strength of association of study characteristics with full publication will be expressed as risk ratios with 95% confidence intervals. We will also explore the possibility of meta-regression analysis to examine the impact of factors specified for subgroup analyses on likelihood of future publication.

#### Subgroup analysis and investigation of potential risk factors for non-publication

Subgroup analyses will be conducted for the following factors depending on availability of data in the included methodological research projects:

• Type of research study (clinical *vs.* animal *vs.* lab research).

• Design of clinical studies (RCT *vs.* CCT *vs.* observational studies *vs.* case reports). If some of these subgroups are not reported separately, we will consider combining groups (RCT *vs.* other designs).

• Funding source (private *vs.* public *vs.* both *vs.* not stated).

• Country of origin of lead investigator (grouped by continent).

• Rank of lead investigator (full professor *vs.* associate/assistant professor *vs.* not stated).

• Sex of lead investigator (male *vs.* female).

• Sample size (below *vs.* above median or mean sample size of included studies).

• Multicenter *vs.* single center study.

• National *vs.* international study.

• English *vs.* non-English publication.

• Significant positive results *vs.* significant negative results *vs.* non-significant results (as defined by primary investigator of methodological research project).

• Clinically relevant results (positive) *vs.* clinically relevant results (negative) *vs.* unclear clinical effect

• Study quality as defined in included primary studies (for example, risk of bias).

• Presentation type (oral *vs.* poster) (abstract aspect only).

• Acceptance for presentation at a scientific conference (abstract aspect only).

We will use definitions applied in included methodological research projects for categorization of, for example, clinically relevant positive/negative results. Further subgroup analyses will be considered if important subgroups of study characteristics arise during the conduct of this systematic review. These exploratory analyses will be clearly labeled as *post-hoc* analyses.

#### Sensitivity analysis

Sensitivity analyses are planned with regard to the four domains used to assess the quality of the methodological research projects (that is, consider only methodological research projects of low study quality) and whether research projects themselves were published as full publications only.

## Discussion

This review seeks to synthesize the growing body of research that is related to non-publication of studies. By examining multiple aspects of biases related to non-publication of studies in one review, we hope to provide an up-to-date, comprehensive picture of this important problem. The findings, including risk factors for non-publication, will serve to raise awareness about the extent of non-publication and the complexity of this issue. In conjunction with results from other work packages of the OPEN project, this review will serve as a foundation for a recommendations workshop. This workshop will enable key members of the biomedical research community (for example, funders, research ethics committees, journal editors) to develop future policies and guidelines to lessen the frequency of non-publication and related biases.

## Appendix

• Appendix A: Search Strategy for OvidSP MEDLINE

• Appendix B: OPEN Consortium

## Appendix A

### A. Search Strategy for OvidSP MEDLINE (search strategy will be adapted for other databases)

#### A.1. Trial registries

1 exp Publishing/sn

2 *publishing/

3 publication bias/

4 selection bias/

5 exp manuscripts as topic/

6 ((data or finding? or information or evidence or study or studies or trial? or paper? or article? or report* or literature or work or manuscript? or abstract* or result?) adj6 (unpublish* or un-publish* or unreport* or un-report* or nonpublish* or non-publish* or nonpublicat* or non-publicat* or (publication? adj3 rate?) or “not publish*”)).ti,ab.

7 (underreport* or under-report* or selective report* or selective publish* or selective publicat* or (final* adj2 (report* or publish* or publicat* or manuscript? or paper? or article?)) or (full? adj2 (report* or publish* or publicat* or manuscript? or paper? or article?)) or (subsequent* adj2 (report* or article? or paper? or publi* or manuscript?)) or (sub-sequent* adj2 (report? or article? or paper? or publi* or manuscript?)) or (complete* adj2 (report* or article? or paper? or publish* or publicat* or manuscript?))).ti,ab.

8 (bias* adj3 (publish* or publicat*)).ti,ab.

9 or/1-8

10 registries/

11 (registry or registries or register?).ti.

12 ((“ClinicalTrials.gov” adj6 regist*) or (Current Controlled Trials adj6 regist*)).ab.

13 or/10-12

14 9 and 13

15 exp animals/ not humans/

16 14 not 15

17 remove duplicates from 16

#### A.2. Research ethics committees

1 exp Publishing/sn

2 *publishing/

3 publication bias/

4 selection bias/

5 exp manuscripts as topic/

6 ((data or finding? or information or evidence or study or studies or trial? or paper? or article? or report* or literature or work or manuscript? or abstract* or result?) adj6 (unpublish* or un-publish* or unreport* or un-report* or nonpublish* or non-publish* or nonpublicat* or non-publicat* or (publication? adj3 rate?) or “not publish*”)).ti,ab.

7 (underreport* or under-report* or selective report* or selective publish* or selective publicat* or (final* adj2 (report* or publish* or publicat* or manuscript? or paper? or article?)) or (full? adj2 (report* or publish* or publicat* or manuscript? or paper? or article?)) or (subsequent* adj2 (report* or article? or paper? or publi* or manuscript?)) or (sub-sequent* adj2 (report? or article? or paper? or publi* or manuscript?)) or (complete* adj2 (report* or article? or paper? or publish* or publicat* or manuscript?))).ti,ab.

8 (bias* adj3 (publish* or publicat*)).ti,ab.

9 or/1-8

10 exp Ethics Committees/

11 exp Ethical Review/

12 clinical protocols/

13 ((institution* adj3 review* adj3 board?) or IRB? or (ethic? adj3 protocol?) or (ethic? adj3 committee?) or ((clinical adj3 protocol?) and ethic*) or ((study adj3 protocol?) and ethic*) or (ethic* adj3 (review* or approv*))).ti,ab.

14 or/10-13

15 9 and 14

16 exp animals/ not humans/

17 15 not 16

18 remove duplicates from 17

#### A.3. Abstracts

1 exp Publishing/sn

2 *publishing/

3 publication bias/

4 selection bias/

5 exp manuscripts as topic/

6 ((data or finding? or information or evidence or study or studies or trial? or paper? or article? or report* or literature or work or manuscript? or abstract* or result?) adj6 (unpublish* or un-publish* or unreport* or un-report* or nonpublish* or non-publish* or nonpublicat* or non-publicat* or (publication? adj3 rate?) or “not publish*”)).ti,ab.

7 (underreport* or under-report* or selective report* or selective publish* or selective publicat* or (final* adj2 (report* or publish* or publicat* or manuscript? or paper? or article?)) or (full? adj2 (report* or publish* or publicat* or manuscript? or paper? or article?)) or (subsequent* adj2 (report* or article? or paper? or publi* or manuscript?)) or (sub-sequent* adj2 (report? or article? or paper? or publi* or manuscript?)) or (complete* adj2 (report* or article? or paper? or publish* or publicat* or manuscript?))).ti,ab.

8 (bias* adj3 (publish* or publicat*)).ti,ab.

9 or/1-8

10 exp animals/ not humans/

11 exp congresses/

12 exp Congresses as Topic/

13 “Abstracting and Indexing as Topic”/

14 abstract?.ti.

15 ((abstract? or proceeding? or poster? or presentation? or presented or presenting or manuscript?) adj6 (congress* or conference? or meeting? or symposi*)).ti,ab.

16 (fate and (abstract? or proceeding? or poster? or presentation? or manuscript?)).ti,ab.

17 or/11-16

18 9 and 17

19 18 not 10

20 remove duplicates from 19

## Appendix B

### B. OPEN Consortium

**Table:** 

Contributor	Participating Institution
Antes, Gerd	German Cochrane Center, Institute of Medical Biometry and Medical Informatics University Medical Center, Freiburg, Germany
Bassler, Dirk	Center for Pediatric Clinical Studies, University Medical Center Tuebingen, Germany
Bertele, Vittorio	Department of Epidemiology, Mario Negri Institute for Pharmacological Research, Italy
Bonfill, Xavier	The Clinical Epidemiology & Public Health Department at the Hospital de la Santa Creu i Sant Pau, Spain
Bouesseau, Marie-Charlotte	World Health Organization, Geneva, Switzerland
Boutron, Isabelle	INSERM U738 research unit, Paris Descartes University, Paris, France
Gallus, Silvano	Department of Epidemiology, Mario Negri Institute for Pharmacological Research, Italy
Garattini, Silvio	Department of Epidemiology, Mario Negri Institute for Pharmacological Research, Italy
Ghassan, Karam	World Health Organization, Geneva, Switzerland
La Vecchia, Carlo	Department of Epidemiology, Mario Negri Institute for Pharmacological Research, Italy
Lang, Britta	German Cochrane Center, Institute of Medical Biometry and Medical Informatics University Medical Center, Freiburg, Germany
Littmann, Jasper	CELLS (Centre for Ethics and Law in Life Sciences), Hannover Medical Scholl, Hannover, Germany
Kleijnen, Jos	Kleijnen Systematic Reviews Ltd., York, United Kingdom
Kulig, Michael	Federal Joint Committee, Berlin, Germany
Malicki, Mario	University of Split School of Medicine, Split, Croatia
Marusic, Ana	University of Split School of Medicine, Split, Croatia
Meerpohl, Joerg	German Cochrane Center, Institute of Medical Biometry and Medical Informatics University Medical Center, Freiburg, Germany
Pardo, Hector	The Clinical Epidemiology & Public Health Department at the Hospital de la Santa Creu i Sant Pau, Spain
Perleth, Matthias	Federal Joint Committee, Berlin, Germany
Ravaud, Philippe	INSERM U738 research unit, Paris Descartes University, Paris, France
Reis, Andreas	World Health Organization, Geneva, Switzerland
Schmucker, Christine	German Cochrane Center, Institute of Medical Biometry and Medical Informatics University Medical Center, Freiburg, Germany
Schwarzer, Guido	German Cochrane Center, Institute of Medical Biometry and Medical Informatics University Medical Center, Freiburg, Germany
Strech, Daniel	CELLS (Centre for Ethics and Law in Life Sciences), Hannover Medical Scholl, Hannover, Germany
Trinquart, Ludovic	INSERM U738 research unit, Paris Descartes University, Paris, France
Urrútia, Gerard	The Clinical Epidemiology & Public Health Department at the Hospital de la Santa Creu i Sant Pau, Spain
Von Elm, Erik	German Cochrane Center, Institute of Medical Biometry and Medical Informatics University Medical Center, Freiburg, Germany Cochrane Switzerland, IUMSP, University Hospital Lausanne, Lausanne, Switzerland
Wager, Elizabeth	Sideview, Princes Risborough, United Kingdom
Wieland, Alexandra	Federal Joint Committee, Berlin, Germany
Wolff, Robert	Kleijnen Systematic Reviews Ltd., York, United Kingdom

## Abbreviations

OPEN: To Overcome Failure to Publish Negative Findings; REC(s): research ethics committee(s); RCT(s): randomized controlled trial(s); CCT(s): controlled clinical trial(s).

## Competing interests

We declare that all authors and contributing members have no competing interests.

## Authors’ contributions

JM is the lead researcher of this project. JM and SP, along with EvE, developed the methodologies of the protocol and led the writing. EM designed the search strategy. DB, GS, and CS contributed significantly to the writing and revision of the protocol. All authors critically revised the protocol and read and approved the final version.
